# Noise-resolution uncertainty principle in classical and quantum systems

**DOI:** 10.1038/s41598-020-64539-7

**Published:** 2020-05-12

**Authors:** Timur E. Gureyev, Alexander Kozlov, David M. Paganin, Yakov I. Nesterets, Harry M. Quiney

**Affiliations:** 1 ARC Centre of Excellence in Advanced Molecular Imaging, the University of Melbourne, Parkville, VIC 3010 Australia; 2 School of Physics and Astronomy, Monash University, Clayton, VIC 3800 Australia; 3 School of Science and Technology, University of New England, Armidale, NSW 2351 Australia; 4 Faculty of Health Sciences, the University of Sydney, Sydney, NSW 2006 Australia; 5 Manufacturing, Commonwealth Scientific and Industrial Research Organisation, Clayton, VIC 3168 Australia

**Keywords:** Quantum optics, Quantum metrology, Information theory and computation, Pure mathematics, Imaging and sensing, Microscopy, Biomedical engineering

## Abstract

We show that the width of an arbitrary function and the width of the distribution of its values cannot be made arbitrarily small simultaneously. In the case of ergodic stochastic processes, an ensuing uncertainty relationship is then demonstrated for the product of correlation length and variance. A closely related uncertainty principle is also established for the average degree of fourth-order coherence and the spatial width of modes of bosonic quantum fields. However, it is shown that, in the case of stochastic and quantum observables, certain non-classical states with sub-Poissonian statistics, such as for example photon number squeezed states in quantum optics, can overcome the “classical” noise-resolution uncertainty limit. This uncertainty relationship, which is fundamentally different from the Heisenberg and related uncertainty principles, can define an upper limit for the information capacity of communication and imaging systems. It is expected to be useful in a variety of problems in classical and quantum optics and imaging.

## Introduction

Various forms of uncertainty principle play an important role in different branches of physics. Well-known examples are the classical diffraction limit in optics^1^ and the Heisenberg uncertainty principle (HUP) in quantum physics^2^. These two principles are related to the same mathematical inequality^3^, as are several other uncertainty relationships in physics^4,5^. Due to these common foundations, the corresponding “phase volumes” are minimized by minimum uncertainty states with similar mathematical form related, for example, to Gaussian or Poisson distributions. A fundamentally different inequality, termed “noise-resolution uncertainty” (NRU), has recently been reported^6,7^. In this context, the “signal” corresponds to the mean value of a measured quantity, with the “noise” being the root-mean-square deviation from the mean. The “resolution” can be associated, for example, with the width of the point-spread function of an optical system or with the width of a bosonic field mode. The NRU inequality is minimized by a type of state related to the Epanechnikov rather than Gaussian distributions^6–8^. This provides a strong indication that, unlike number-phase and many other uncertainty relationships in physics, the NRU (which formally corresponds to number-position uncertainty) cannot be derived from the HUP or similar relationships.

As shown below, in its most general form, the NRU states that the width of a function and the width of the distribution of its values cannot be simultaneously made arbitrarily narrow. When applied to stochastic distributions, the NRU implies that a distribution, such as a detected spatial distribution of identical particles, cannot be made arbitrarily spatially narrow and have arbitrary high signal-to-noise ratio (SNR) at the same time, if the total mean number of particles in the system is kept constant. This implies a fundamental trade-off between the (spatial) resolution and the SNR of a distributed classical or quantum measurement. This may seem counter-intuitive at first in view of the obvious existence of Dirac-delta-like distributions, which may be perceived as having both “perfect” localization and arbitrarily high SNR. Nevertheless, the applicability of the NRU principle to such distributions is demonstrated below.

We show in this paper that the NRU principle can be expressed in a general form which is different from and has broader applicability compared to our earlier results^6,7,9^. Related results have been also reported previously in the field of classical imaging^10–12^, quantum optics^13–19^ and quantum metrology^20,21^. In general, the existence of a noise-resolution trade-off has long been recognised^22^, and some related notions such as, for example, the Detective Quantum Efficiency (DQE) in detector technology, have been introduced and successfully used in practice^23^. The existence and precise quantitative form of NRU-related trade-offs are likely to be important in many fields of science and technology, from medical imaging and semiconductor manufacturing to quantum computing and experiments with ultra-cold atoms. Therefore, it appears useful to establish a general and precise expression for the NRU in a transparent form that can be readily related to typical settings of different physical measurements. This objective is addressed below. Following a general mathematical description, we demonstrate how the proposed approach can be applied to stochastic processes, where it provides a relationship between correlation length and variance. As another application, we derive a NRU relationship in quantum optics. Here, in particular, the present paper contains a complementary result to the one reported earlier^9^ which demonstrated an NRU-type inequality for the electric energy operator associated with the quantised electromagnetic field. The latter result relied on the existence of an absolute positive lower limit for the variance of electric energy, determined by the vacuum fluctuations^24^. As mentioned^9^, this approach could not be extended to the more practically relevant photon number operator, because its variance can be zero in Fock states. The approach developed in the present paper allows us to overcome this difficulty and obtain NRU-type relations for certain correlation functions in quantum optics. In this context, we then give an example of a quantum system that can defeat the classical limit for the product of the variance and the width of a single-mode field. Interestingly, it appears that even for such specially designed states, the NRU limit can be exceeded only by a small fraction which rapidly goes to zero when the mean number of photons in the system becomes large. It is therefore likely that the NRU may impose effective bounds on the precision of certain quantum measurements and quantum information capacity^4,20,21^.

## Results

### General relationship between the width and the variance of a function

**Figure 1:**
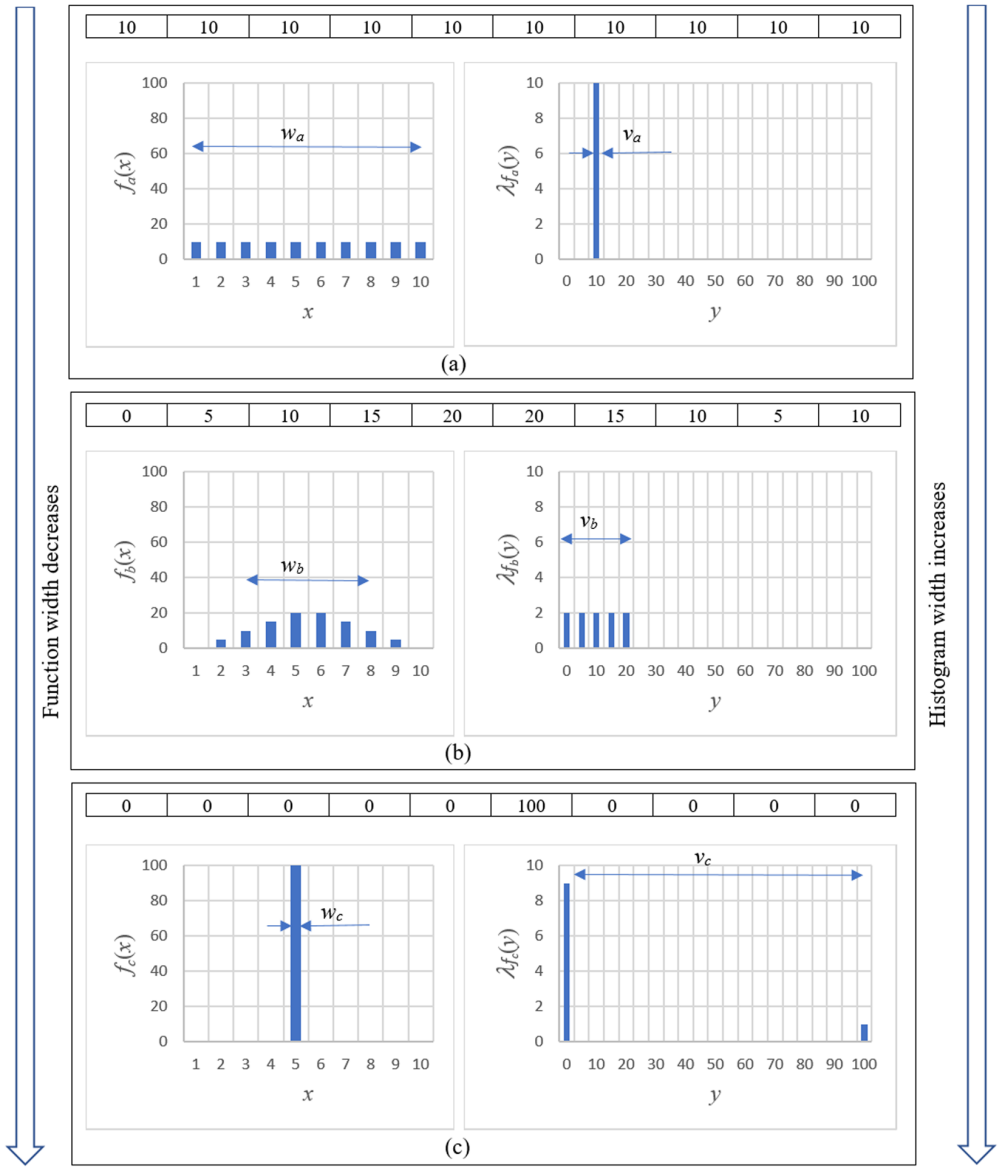
Relationship between the width *w* of a function *f* and the width *v* of its associated histogram ${\lambda }_{f}$. (**a**) Very wide function, very narrow histogram. (**b**) Narrower function, broader histogram. (**c**) Very narrow function, very broad histogram.

Figure 1 illustrates, in an informal manner, the relationship between the width *w* of a function *f*, and the width *v* of its associated histogram ${\lambda }_{f}$. Here, for the purposes of illustration, we consider *N* = 100 imaging quanta (e.g. photons, neutrons, electrons etc.) that are collected by a one-dimensional position-sensitive detector composed of ten adjacent pixels. Figure 1(a) shows the case where all imaging quanta are uniformly distributed amongst the pixels, giving the intensity function *f*_*a*_(*x*) as a function of pixel coordinate *x*, that has a width *w*_*a*_ filling the entire imaging domain; the associated histogram ${\lambda }_{{f}_{a}}$ has a narrow single-bin width *v*_*a*_.

In Fig. 1(b), the function is made narrower, to give *f*_*b*_(*x*) whose width *w*_*b*_ is narrower than *w*_*a*_, but whose histogram ${\lambda }_{{f}_{b}}$ has a width *v*_*b*_ that is broader than *v*_*a*_. A similar trend is seen in passing to Fig. 1(c). The key point, here, is that a reciprocal relationship exists between *v* and *w* - decreasing the width *w* of a function leads to an increase in the width *v* of the associated histogram, and vice versa.

**Figure 2:**
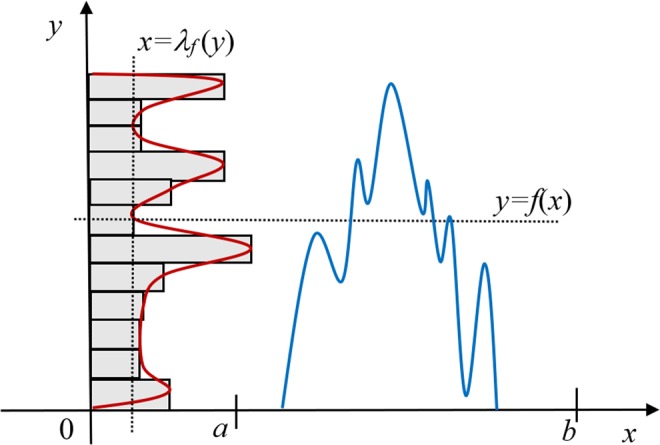
Graphical representation of a “generic” one-dimensional function and its histogram.

The above construction motivates the following more formal treatment. Consider an arbitrary measurable (in the sense of Radon) function $f(x)\ge 0$ of real variable *x*, such that $f(x)=0$ outside some finite interval Ω = [*a*, *b*]. We study the one-dimensional case first for simplicity, but use notation, such as $|\Omega |\equiv b-a$ for the length of $\Omega$, that allows easy translation of the main results to higher dimensions later. The (normalized) histogram of $f(x)$ can be defined in a similar way to the probability density function (PDF). Firstly, consider a function ${L}_{f}(y)$ equal to the fraction (relative area) of all points *x* in Ω at which $f(x)\le y$, i.e.

1 $${L}_{f}(y)=|\Omega {|}^{-1}{\int }_{\Omega }\theta [y-f(x)]dx,$$

where $\theta (x)$ is the Heaviside step-function which is equal to zero, when $x < 0$, and equal to 1, when $x\ge 0$. The normalized histogram function is then defined as the (generalized) derivative of ${L}_{f}(y)$, that is

2 $${\lambda }_{f}(y)\equiv d{L}_{f}(y)/dy=|\Omega {|}^{-1}{\int }_{\Omega }\delta [y-f(x)]dx,$$

where $\delta (x)$ is the Dirac delta (Fig. 2). As ${L}_{f}(y)$ is a monotonically non-decreasing function of *y*, ${\lambda }_{f}(y)$ is non-negative everywhere and $\int {\lambda }_{f}(y)dy={L}_{f}(\,+\,\infty )-{L}_{f}(\,-\,\infty )=1$. Therefore, ${\lambda }_{f}(y)$ can be viewed as a PDF associated with the “random variable” $y=f(x)$. Such an interpretation can be literal for example, if $f(x)$ is a sample of a spatially-ergodic stochastic process $\{f(x)\}$.

A useful result involving the histogram of a function is the following equation for mean values:

3 $$|\Omega {|}^{-1}{\int }_{\Omega }g[f(x)]dx=\int g(y){\lambda }_{f}(y)dy,$$

where *g*(*y*) is an arbitrary function selected from a suitably broad functional class. For example, when $g(y)=y$, we have

4 $$\bar{f}\equiv |\Omega {|}^{-1}{\int }_{\Omega }f(x)dx=\int y{\lambda }_{f}(y)dy.$$

For this reason, Eq. (3) is sometimes called “the law of the unconscious statistician”^25^, because it states an intuitive fact that a mean value of a function can be equally evaluated either by averaging its “trial” values, or by integrating over the relevant PDF. In the present setting, Eq. (3) is easily proved by substituting ${\lambda }_{f}(y)=|\Omega {|}^{-1}{\int }_{\Omega }\delta [f(x)-y]dx$ on the right-hand side of Eq. (3) and changing the order of integration.

We define the width of a function $f(x)\ge 0$ in the usual way via its standard deviation:

5 $${\Delta }_{x}[f]\equiv {[{\int }_{\Omega }{(x-\bar{x})}^{2}f(x)dx/{\int }_{\Omega }f(x)dx]}^{1/2},$$

where

6 $$\bar{x}\equiv {\int }_{\Omega }xf(x)dx/{\int }_{\Omega }f(x)dx.$$

**Figure 3:**
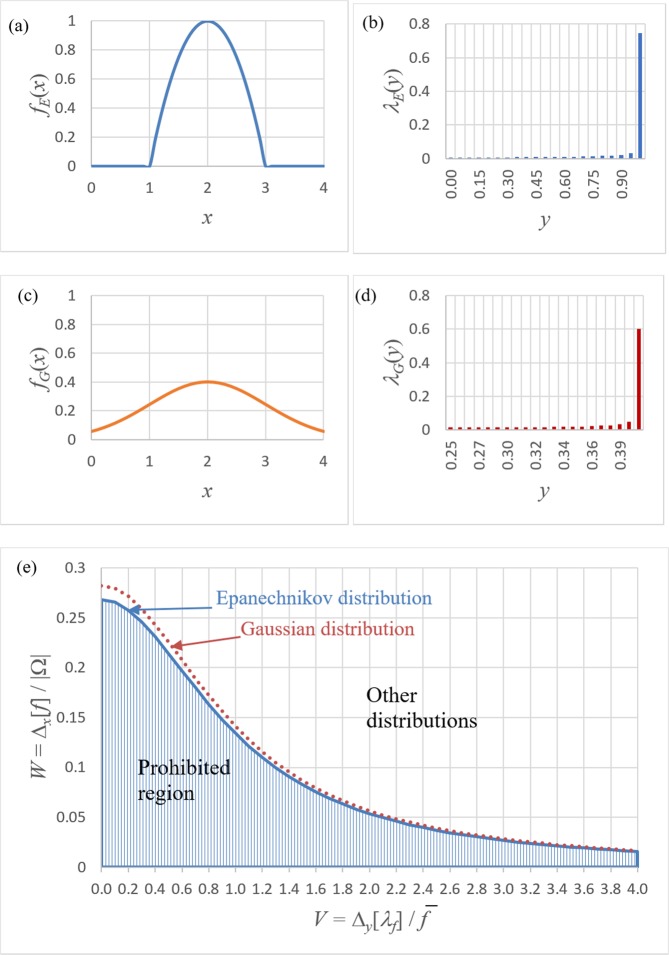
Illustration of noise-resolution uncertainty principle in Eq. (7). (**a**) Epanechnikov distribution in Ω = [0, 4]; (**b**) Histogram of the Epanechnikov distribution; (**c**) Truncated Gaussian distribution; (**d**) Histogram of the truncated Gaussian distribution; (**e**) Dependence of the relative width of a distribution on the relative width of its histogram for various distributions.

We would like to quantify and prove the following hypothesis whose intuitive content is evident in Fig. 1 (see also the first paragraph of this section): when $f(x)$ becomes narrow, its histogram ${\lambda }_{f}(y)$ becomes broad, and vice versa. In other words, it is impossible to make a function and its histogram arbitrarily narrow, simultaneously. In accordance with this hypothesis, one could try to show that the product of the widths of $f(x)$ and ${\lambda }_{f}(y)$, i.e. ${\Delta }_{x}[f]{\Delta }_{y}[{\lambda }_{f}]$, is always larger than some positive value. Unfortunately, this is clearly not true for finite intervals $\Omega =[a,b]$, because the width of the histogram is zero for constant functions $f(x)=c$, while the width of such functions is finite. However, it turns out that the following closely related inequality does indeed hold for any non-negative function *f*, for which the involved integrals are well defined:

7 $${\Delta}_{x}[f]({\Delta}_{y}^{2}[{\lambda}_{f}]+{\bar{f}}^{2})\ge {C{\prime}_{1}}{|}\Omega {|}{\bar{f}}^{2},$$

where ${C{\prime} }_{1}\cong 0.27$ is a dimensionless constant which is precisely defined below. Note that Eq. (7) holds for constant functions $f(x)=c$, because ${\Delta }_{x}[c]/|\Omega |=1/(2\sqrt{3})\cong 0.29 > {C{\prime} }_{1}$ for any constant value *c* > 0. Moreover, Eq. (7) is exact; it becomes an equality for certain non-negative functions, such as the Epanechnikov function

8 $${f}_{E}(x)=C{[1-{(2x-a{\prime} -b{\prime} )}^{2}/(a{\prime} -b{\prime} )]}_{+},$$

where *C* is an arbitrary positive constant, $a\le a{\prime}  < b{\prime} \le b$ and the subscript “+” denotes that the function is equal to zero at points where the expression inside the square brackets is negative. See Fig. 3(a) for a sketch of the functional form of the Epanechnikov distribution, together with the associated histogram in Fig. 3(b). For comparison, the truncated Gaussian distribution is shown in Fig. 3(c), together with its associated histogram in Fig. 3(d).

A general proof of Eq. (7) follows from the identity

$${\Delta }_{y}^{2}[{\lambda }_{f}]+{\bar{f}}^{2}=\int {y}^{2}{\lambda }_{f}(y)dy=|\Omega {|}^{-1}{\int }_{\Omega }{f}^{2}(x)dx$$

that can be obtained from Eq. (3) with $g(y)={y}^{2}$, and the one-dimensional (*d* = 1) version of the mathematical inequality:

9 $$\parallel f{\parallel }_{2}^{2}{\Delta }_{x}^{d}[f]\ge {C{\prime} }_{d}\parallel f{\parallel }_{1}^{2}.$$

Here $\parallel f{\parallel }_{p}\equiv {(\int |f(x){|}^{p}dx)}^{1/p}$ for any *p* > 0,

10 $${C{\prime} }_{d}\equiv {[d/(4\pi )]}^{d/2}{C}_{d},$$

where

11 $${C}_{d}={2}^{d}\Gamma (d/2)d(d+2)/{(d+4)}^{d/2+1}$$

is the Epanechnikov constant^8^ and Γ denotes the gamma function. The constants *C*_*d*_ are of the order of 1 for low dimensions *d*; in particular, ${C}_{1}=(6/5)\sqrt{\pi /5}\cong 0.95$ and hence ${C{\prime} }_{1}={C}_{1}/(2\sqrt{\pi })=3/(5\sqrt{5})\cong 0.27$. Equation (9) is invariant with respect to multiplicative scaling of $f(x)$ and its argument, i.e. Equation (9) does not change if we replace $f(x)$ with $\alpha f(\beta x)$, where *α* and *β* are arbitrary real positive constants. This scaling bi-invariance of Eq. (9) is the same as that of the HUP^3,6^.

Before continuing, we make some interpretive remarks. The form of the noise-resolution uncertainty principle, given in Eq. (7), is sketched in Fig. 3(e). This shows the scaled width $W={\Delta }_{x}[f]/\Omega$ of any Radon-measurable function *f*(*x*), versus the associated scaled width $V={\Delta }_{y}[{\lambda }_{f}]/\bar{f}$ of the histogram of *f*(*x*). The shaded region outlines the prohibited values for $(V,W),$ with the noise-resolution trade-off being evident in the fact that decreasing the function width *W* forces the minimum permitted scaled histogram width *V* to increase. This quantifies the trend that was evident, from an informal perspective, in Fig. 1. Note that we have denoted the horizontal axis of Fig. 3(e) by the symbol *V*, since this quantity may be taken as defining the Michelson-type “visibility” of the function *f*(*x*) to be the histogram width ${\Delta }_{y}[{\lambda }_{f}]$ divided by the mean value $\bar{f}$of *f*(*x*). The Epanechnikov (inverted parabola) distribution ${f}_{E}(x)$, shown in the left panel of Fig. 3(a) together with the associated histogram ${\lambda }_{E}(y)$ in Fig. 3(b), achieves the minimum *W* for any given *V*. This Epanechnikov distribution corresponds to the solid line, bounding the shaded region from above, in Fig. 3(e). For comparison, the Gaussian function ${f}_{G}(x)$ in the left panel of Fig. 3(c), together with the associated histogram ${\lambda }_{G}(y)$ in Fig. 3(d), is non-optimal in the sense of corresponding to the non-lower-bound dotted line in Fig. 3(e). This illustrates a previously-mentioned point, that while Gaussian distributions minimise the position-momentum uncertainty product given by the Heisenberg uncertainty principle, such distributions are not optimal from a noise-resolution perspective. Conversely, Epanechnikov distributions are optimal from the perspective of the trade-off between noise and resolution, but are non-optimal with regard to the trade-off between position (width) and momentum. Because the functional $\{{\Delta }_{x}[f]/|\Omega |\}\{{\Delta }_{y}^{2}[{\lambda }_{f}]/{\bar{f}}^{2}+1\}$ is invariant with respect to rescaling of the width or the height of the function *f* ^9^, one obtains that $W(V)={C}_{f}/(1+{V}^{2})$ for any given function type. This implies, in particular, that the curves $W(V)$ always asymptotically converge to zero when $V\to \infty$.

Consider now the “variance” of a function defined in the conventional way:

12 $${\rm{Var}}[f]\equiv |\Omega {|}^{-1}{\int }_{\Omega }{(f-\bar{f})}^{2}(x)dx=\int {(y-\bar{y})}^{2}{\lambda }_{f}(y)dy,$$

where we have used Eq. (3) with $g(y)={(y-\bar{y})}^{2}$. Equation (12) implies, in particular, that the variance of a function is equal to the square of the width of its histogram:

13 $${\rm{Var}}[f]={\Delta }_{y}^{2}[{\lambda }_{f}].$$

Hence, Eq. (7) can be re-written as

14 $$\left(\frac{{\rm{Var}}[f]}{{(\bar{f})}^{2}}+1\right)\frac{{\Delta }_{x}[f]}{|\Omega |}\ge {C{\prime} }_{1}.$$

It is instructive to consider the NRU Eq. (14) for Dirac-delta type functions. If $f(x)=\delta (x),$ then ${\rm{Var}}[f]$ cannot be formally evaluated, because the square of the delta function is not defined in the space of generalized functions. Consider, however, an area ${\Omega }_{b}\equiv [\,-\,b,b]$ and the set of Gaussians

15 $${f}_{\sigma }(x)={(2\pi )}^{-1/2}{\sigma }^{-1}\,\exp [\,-\,|x{|}^{2}/(2{\sigma }^{2})],$$

with $\sigma \ll b$, which approximate $\delta (x)$ when $\sigma \to 0$. It is easy to calculate that ${\bar{f}}_{\sigma }\cong 1/(2b),$ ${\rm{Var}}[{f}_{\sigma }]\cong 1/(4b\sigma \sqrt{\pi })$, ${\Delta }_{x}[{f}_{\sigma }]\cong \sigma$, and hence in the left-hand side of Eq. (14) we have

16 $$\{1+{\rm{Var}}[{f}_{\sigma }]/{({\bar{f}}_{\sigma })}^{2}\}{\Delta }_{x}[{f}_{\sigma }]/(2b)\cong {(2\sqrt{\pi })}^{-1}+\sigma /(2b).$$

This value tends to the constant ${(2\sqrt{\pi })}^{-1}\cong 0.28 > {C{\prime} }_{1}$, when $\sigma \to 0$, and, in particular, the NRU does not decrease below this positive value when the Gaussians approach the Dirac delta function.

### Noise-resolution uncertainty for stochastic processes

**Figure 4:**
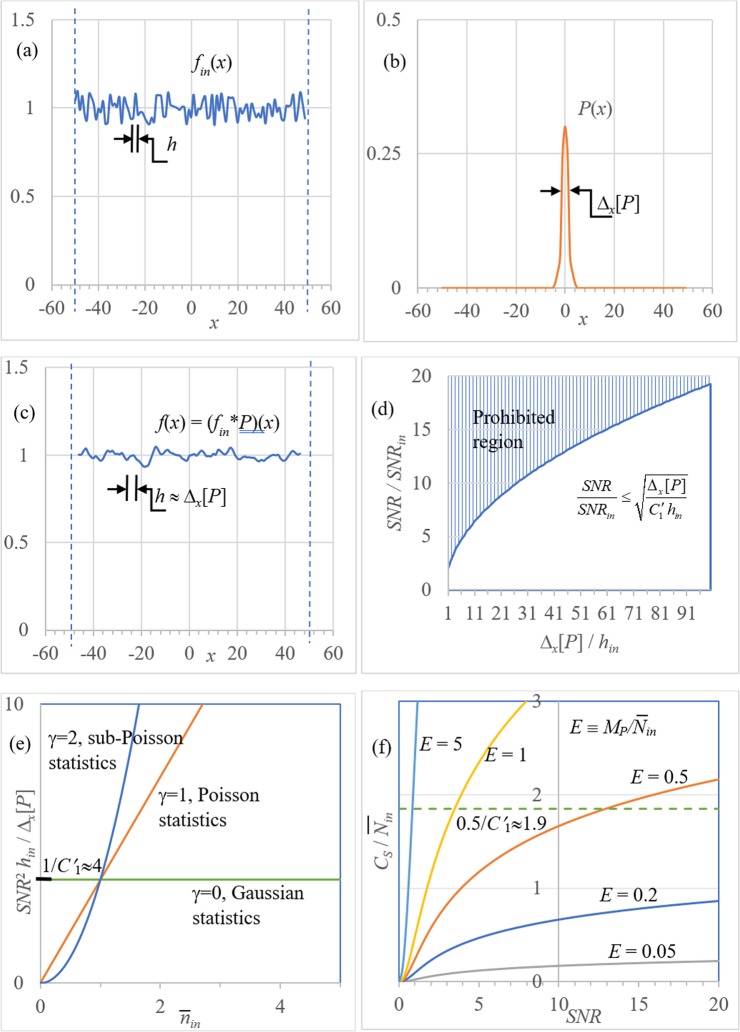
(**a**) Input ergodic stochastic distribution *f*_*in*_(*x*); (**b**) point-spread function *P*(*x*); (**c**) output ergodic stochastic function *f*(*x*) = (*f*_*in*_ * P)(*x*); (**d**) illustration of Eq. (22); (**e**) illustration of Eq. (23); (**f**) illustration of Eq. (24).

Let us consider an application of the above result to ergodic stochastic distributions (spatial stochastic processes). See Fig. 4(a). Here it is convenient to work with symmetric domains ${\Omega }_{b}=[\,-\,b,b]$. To keep the notation consistent with that used above, instead of considering the proper limits of various quantities at $b\to \infty$ as required in the theory of stochastic processes, we simply assume that *b* is sufficiently large. For example, the autocorrelation function will be defined as

17 $${\varGamma }_{f}(x)=|{\Omega }_{b}{|}^{-1}{\int }_{{\Omega }_{b}}f(x+x{\prime} )f(x{\prime} )dx{\prime} ,$$

implicitly assuming that *b* is so large that contribution from the “tails” of the integral at $|x|\ge b$ is negligible for all considered distributions $f(x)$.

Because ergodic distributions $f(x)$ are always statistically stationary^26^, the notion of width does not make sense for them. However, the width of ${\Gamma }_{f}(x)$, which is the correlation length, is well defined. We consider here a frequently encountered case of “transmission” through a smearing information channel, where $f(x)$ is a convolution of an “input” distribution ${f}_{in}(x)$ as sketched in Fig. 4(a), with a deterministic point-spread function $P(x)$ (Fig. 4(b)),

18 $$f(x)=({f}_{in}\ast P)(x)=\int {f}_{in}(x-x{\prime} )P(x{\prime} )dx{\prime} ,$$

as for example, in a typical case of a photon flux measured by a position-sensitive area detector (see Fig. 4(c)). For each distribution, we also consider the corresponding “noise” distribution $\tilde{f}(x)=f(x)-\bar{f}$. When the noise ${\tilde{f}}_{in}(x)$ in the input distribution is essentially uncorrelated, its correlation length is very small and the correlation length $h\approx {\Delta }_{x}[P]$ of $\tilde{f}={\tilde{f}}_{in}\ast P$ is determined by the width of $P(x)$ (cf. Fig. 4(a–c)). Under these conditions, we obtain an analogue of the NRU for the stochastic distribution $f={f}_{in}\ast P$ by applying Eq. (9) to $P(x).$ Firstly, note that

19 $$\bar{f}=|{\Omega }_{b}{|}^{-1}{\int }_{{\Omega }_{b}}\int {f}_{in}(x-x{\prime} )P(x{\prime} )dx{\prime} dx\cong {\bar{f}}_{in}\parallel P{\parallel }_{1}.$$

It is also easy to verify that ${\Gamma }_{\tilde{f}}(x)=({\Gamma }_{{\tilde{f}}_{in}}\ast {P}_{2})(x)$, where ${P}_{2}(x)\equiv \int P(x+x{\prime} )P(x{\prime} )dx{\prime}$ is the autocorrelation of $P(x)$. Therefore,

20 $${\rm{Var}}[f]={\Gamma }_{\tilde{f}}(0)=\int {\Gamma }_{{\tilde{f}}_{in}}(-x{\prime} ){P}_{2}(x{\prime} )dx{\prime} \cong {\Gamma }_{{\tilde{f}}_{in}}(0){h}_{in}{P}_{2}(0)={\rm{Var}}[{f}_{in}]{h}_{in}\parallel P{\parallel }_{2}^{2},$$

where we have used the fact that ${\tilde{f}}_{in}(x)$ is uncorrelated and hence ${\Gamma }_{{\tilde{f}}_{in}}(y)$ is very narrow (we denoted its width by *h*_*in*_, which could correspond to the pixel size in the case of a photon-counting detector). Combining the above results, we obtain ${\rm{Var}}[f]/{(\bar{f})}^{2}={h}_{in}\parallel P{\parallel }_{2}^{2}/(\parallel P{\parallel }_{1}^{2}\,{{\rm{SNR}}}_{in}^{2})$, where ${{\rm{SNR}}}_{in}\equiv {\bar{f}}_{in}/{\sigma }_{in}$. Multiplying this equation by Δ_*x*_[*P*] and applying Eq. (9) with *d* = 1 for $P(x)$, i.e. $\parallel P{\parallel }_{2}^{2}{\Delta }_{x}[P]\ge {C{\prime} }_{1}\parallel P{\parallel }_{1}^{2}$, leads to the following analogue of Eq. (14):

21 $$\frac{{\rm{Var}}[f]}{{(\bar{f})}^{2}}\frac{{\Delta }_{x}[P]}{|{\Omega }_{b}|}\ge \frac{{C{\prime} }_{1}}{{M}_{in}\,{{\rm{SNR}}}_{in}^{2}},$$

where ${M}_{in}\equiv |{\Omega }_{b}|/{h}_{in}$ is the number of autocorrelation lengths of the input distribution that fit into Ω_*b*_. Consequently, we obtain

22 $${M}_{P}\,{{\rm{SNR}}}^{2}\le {({C{\prime} }_{1})}^{-1}{M}_{in}\,{{\rm{SNR}}}_{in}^{2},$$

where ${M}_{P}\equiv \,|{\Omega }_{b}|/{\Delta }_{x}[P]$. The difference in form between Eq. (14) and Eq. (21) includes the absence of the additive term 1 inside the brackets on the left-hand side and the presence of the term ${M}_{in}\,{{\rm{SNR}}}_{in}^{2}$ in the denominator on the right-hand side. If ${f}_{in}$ has large SNR_*in*_ and/or large *M*_*in*_, then the right-hand side of Eq. (21) can be close to zero, in which case Eq. (21) does not impose any substantial lower limit on the product of the 1/SNR^2^ and the width of *P*(*x*). We see below that a similar situation can occur for quantum fields.

Returning to Eq. (22), this expression may be re-arranged to give the inequality for SNR/SNR_*in*_ that is plotted in Fig. 4(d). The vertical axis of Fig. 4(d) may be viewed as a “signal-to-noise-ratio gain factor” which quantifies how much larger the SNR of the PSF-smoothed signal *f*(*x*) is, compared to the unsmoothed signal *f*_*in*_(*x*). The greater the degree of smoothing, the further one moves along the horizontal axis of Fig. 4(d), and the greater the maximum degree of SNR improvement (at the expense of coarsened spatial resolution). This form of the noise-resolution inequality makes the intuitively reasonable statement that binning over larger effective pixels will reduce noise, and hence increase SNR, at the expense of coarsened spatial resolution.

In relation to imaging problems, note that the structure of Eq. (22) is reminiscent of the Detective Quantum Efficiency (DQE) at zero frequency, which describes the efficiency of a detector and is usually equal to the ratio of the squared SNR in output and input signals^23^. A special case of Eq. (22) corresponds to ${f}_{in}(x)$ being an uncorrelated distribution with mean number of photons ${\bar{n}}_{in}$ collected in each detector pixel and ${{\rm{SNR}}}_{in}^{2}={\bar{n}}_{in}^{\gamma }$, where $0\le \gamma  < 1$ in the case of super-Poissonian statistics ($\gamma =0$ for Gaussian statistics), $\gamma =1$ for Poissonian statistics and $1 < \gamma  < \infty$ for sub-Poissonian statistics^27^. Here, Eq. (22) becomes^9^

23 $$\frac{{{\rm{SNR}}}^{2}}{{\Delta }_{x}[P]}\le \frac{{\bar{n}}_{in}^{\gamma }}{{C{\prime} }_{1}{h}_{in}}.$$

See Fig. 4(e). Equation (23) with $\gamma =1$ expresses a typical form of NRU in imaging^6^, where, at a fixed incident photon fluence ${F}_{in}={\bar{n}}_{in}/{h}_{in}$, the ratio of the squared SNR to spatial resolution cannot be increased beyond a certain absolute limit. More generally, if ${\bar{n}}_{in} > 1$ and $\gamma \gg 1$, ${\bar{n}}_{in}^{\gamma }$ can in principle be very large and the SNR can be essentially decoupled from the correlation length of a stochastic distribution, at a fixed incident fluence (radiation dose) level. A similar phenomenon is also observed in the context of quantum field theory, as demonstrated below. This can be advantageous in imaging of radiation-sensitive samples^6,20^.

We note that the above results have direct implications for the information capacity of imaging systems^11,12,28^ and possibly for quantum information capacity as well^17–19^. In particular, Eq. (22) provides an upper bound for the Shannon information capacity *C*_*S*_ of an imaging system with *M*_*P*_ independent channels (effective pixels) and Poissonian noise statistics, ${{\rm{SNR}}}_{in}^{2}={\bar{n}}_{in}$:

24 $${C}_{S}\cong 0.5{M}_{P}\,{\log }_{2}(SN{R}^{2}+1)\le 0.5{M}_{P}SN{R}^{2}\le {\bar{N}}_{in}/(2{C{\prime} }_{1}),$$

where ${\bar{N}}_{in}\equiv {M}_{in}{\bar{n}}_{in}$ denotes the mean total number of photons used for imaging. Equation (24) implies that the Shannon information capacity per photon is limited from above by an absolute constant ${(2{C{\prime} }_{1})}^{-1}\cong 1.9$. This limit from above on the Shannon information capacity per photon (${C}_{S}/{\bar{N}}_{in}$) is shown as the dashed horizontal line in Fig. 4(f). The curves in this figure correspond to ${C}_{S}/{\bar{N}}_{in}$ versus SNR for five different values of *E*, where $E={M}_{P}/{\bar{N}}_{in}$ is the ratio of (i) the number of independent channels (effective pixels) to (ii) the mean total number of photons. When the number of effective pixels per photon *E* has the relatively small values of *E* = 0.05 or *E* = 0.2, which corresponds to a scenario in which there are many photons per effective pixel, the classic Shannon-information-per-photon curves are not constrained by the noise-resolution limit given by the dashed line, for the plotted range of SNR values from 0 to 20. Stated differently, noise does not affect resolution when there are many photons per effective pixel. However, for the same plotted range of SNR values, the classic Shannon-information-per-photon curves are constrained by the noise-resolution limit, when the number of effective pixels per photon takes the values *E* = 0.5, 1, 5; this corresponds to the case where there are few photons per effective pixel, which is precisely the domain in which noise affects resolution: one cannot ignore the implications of noise-resolution uncertainty in such an imaging regime.

### Noise-resolution uncertainty in quantum field theory

Thus far, all of our discussions have used a classical-optics formalism. However, in the dilute-illumination case where the mean number of photons per pixel, ${\bar{n}}_{in}$, becomes small, the quantum nature of light will become important. Hence, for example, Eq. (23) will become less reliable in this regime where quantum effects become more signicant (i.e. when ${\bar{n}}_{in} < 1$; cf. Fig. 4(e)). In this regime, the inevitable contribution of detector and/or source statistics cannot be ignored, motivating a quantum field-theoretic treatment of noise-resolution uncertainty to which we now turn.

Consider an analogue of the NRU Eq. (14) in quantum electrodynamics (QED). For a single-mode electric field,

25 $${E}_{k}({\bf{r}},t)={E}_{k}^{(+)}({\bf{r}},t)+{E}_{k}^{(-)}({\bf{r}},t),\,{E}_{k}^{(+)}({\bf{r}},t)=i{(\hslash {\omega }_{k}/2)}^{1/2}{u}_{k}({\bf{r}})\,\exp (\,-\,i{\omega }_{k}t){\hat{a}}_{k},$$

inside a cube ${\Omega }_{L}$ with side length *L* centred at ${\bf{r}}=0$ in 3D space, where $\hslash$ is the reduced Planck constant, ${\omega }_{k}$ is the angular frequency of the mode, ${u}_{k}({\bf{r}})$ is the mode function, ${E}^{(-)}({\bf{r}},t)={[{E}^{(+)}({\bf{r}},t)]}^{\dagger }$ is the Hermitian conjugate of ${E}^{(+)}({\bf{r}},t)$, ${\hat{a}}_{k}$ and ${\hat{a}}_{k}^{\dagger }$ are the photon annihilation and creation operators for the *k*th mode, respectively^27^.

If *ρ* is a density operator and $\langle {n}_{k}\rangle$ is the mean number of photons in the mode, then

26 $${\rm{Tr}}[\rho {\hat{a}}_{k}^{\dagger }{\hat{a}}_{k}]=\langle {n}_{k}\rangle ,$$

27 $${\rm{Tr}}[\rho {\hat{a}}_{k}^{\dagger }{\hat{a}}_{k}^{\dagger }{\hat{a}}_{k}{\hat{a}}_{k}]={\rm{Tr}}[\rho {({\hat{a}}_{k}^{\dagger }{\hat{a}}_{k})}^{2}]-{\rm{Tr}}[\rho {\hat{a}}_{k}^{\dagger }{\hat{a}}_{k}]=\langle {n}_{k}^{2}\rangle -\langle {n}_{k}\rangle ,$$

where we have used the commutation relation $[{\hat{a}}_{k},{\hat{a}}_{k}^{\dagger }]=1$^27^. Therefore, the coherence functions of the second and fourth order^27,29^, in the special case of single-point measurements, can be expressed as

28 $${G}_{1}({\bf{r}})={\rm{Tr}}[\rho {E}_{k}^{(-)}({\bf{r}},t){E}_{k}^{(+)}(r,t)]=\langle {n}_{k}\rangle (\hslash {\omega }_{k}/2){f}_{k}({\bf{r}}),$$

29 $$\begin{array}{rcl}{G}_{2}({\bf{r}}) & \equiv  & {\rm{Tr}}[\rho {E}_{k}^{(-)}({\bf{r}},t){E}_{k}^{(-)}({\bf{r}},t){E}_{k}^{(+)}({\bf{r}},t){E}_{k}^{(+)}({\bf{r}},t)]\\  & = & (\langle {n}_{k}^{2}\rangle -\langle {n}_{k}\rangle ){(\hslash {\omega }_{k}/2)}^{2}{f}_{k}^{2}({\bf{r}}),\end{array}$$

where ${f}_{k}({\bf{r}})\equiv |{u}_{k}({\bf{r}}){|}^{2}$. For any function *G*(**r**) defined in Ω_*L*_ we can consider the spatial average

30 $$\bar{G}\equiv |{\Omega }_{L}{|}^{-1}\,{\int }_{{\Omega }_{L}}\,G({\bf{r}})d{\bf{r}},\,|{\Omega }_{L}|={L}^{3},$$

and the width

31 $${\Delta }_{r}[G]={[{\int }_{{\Omega }_{L}}|{\bf{r}}-\overline{{\bf{r}}}{|}^{2}G({\bf{r}})d{\bf{r}}/{\int }_{{\Omega }_{L}}G({\bf{r}})d{\bf{r}}]}^{1/2},$$

where $\overline{{\bf{r}}}$ is the mean value of **r** with respect to the PDF $G({\bf{r}})/{\int }_{{\Omega }_{L}}\,G({\bf{r}})d{\bf{r}}$. This allows us to introduce the quantities

32 $${\overline{G}}_{1}=\langle {n}_{k}\rangle \,(\hslash {\omega }_{k}/2){\bar{f}}_{k},$$

33 $${\overline{G}}_{2}=[\langle {n}_{k}^{2}\rangle -\langle {n}_{k}\rangle ]{(\hslash {\omega }_{k}/2)}^{2}\overline{{f}_{k}^{2}},$$

and to consider the following analogue of the functional on the left-hand side of the NRU Eq. (14):

34 $$F[E]\equiv \frac{{\overline{G}}_{2}}{{({\overline{G}}_{1})}^{2}}\frac{{({\Delta }_{r}[{G}_{1}])}^{3}}{|{\Omega }_{L}|}=\left[\frac{{\overline{G}}_{2}-{({\overline{G}}_{1})}^{2}}{{({\overline{G}}_{1})}^{2}}+1\right]\frac{{({\Delta }_{r}[{G}_{1}])}^{3}}{|{\Omega }_{L}|}.$$

It is easy to verify that

35 $$F[E]=\left(\frac{\langle {n}_{k}^{2}\rangle -\langle {n}_{k}\rangle }{{\langle {n}_{k}\rangle }^{2}}\right)\frac{{\int }_{{\Omega }_{L}}\,{f}_{k}^{2}({\bf{r}})d{\bf{r}}}{{({\int }_{{\Omega }_{L}}{f}_{k}({\bf{r}})d{\bf{r}})}^{2}}{\left[\frac{{\int }_{{\Omega }_{L}}|{\bf{r}}-\bar{{\bf{r}}}{|}^{2}{f}_{k}({\bf{r}})d{\bf{r}}}{{\int }_{{\Omega }_{L}}{f}_{k}({\bf{r}})d{\bf{r}}}\right]}^{3/2}.$$

Applying Eq. (9) with *d* = 3 to ${f}_{k}({\bf{r}})$ in Eq. (35), we obtain the following form of the NRU:

36 $$\left[\frac{{\overline{G}}_{2}-{({\overline{G}}_{1})}^{2}}{{({\overline{G}}_{1})}^{2}}+1\right]\frac{{\Delta }_{r}^{3}[{G}_{1}]}{|{\Omega }_{L}|}\ge {C{\prime} }_{3}\left(1+\frac{Q}{\langle {n}_{k}\rangle }\right),$$

where $${C{\prime} }_{3}={[3/(4\pi )]}^{3/2}{C}_{3}={(3/7)}^{1/2}45/(98\pi )\cong 0.1$$ and

37 $$Q\equiv (\langle {(\Delta {n}_{k})}^{2}\rangle /\langle {n}_{k}\rangle )-1$$

is the Mandel *Q* parameter^29,30^. For Poissonian statistics, corresponding to coherent states, we have *Q* = 0 and Eq. (36) transforms back into the “classical” form of the NRU. In non-classical quantum states with sub-Poissonian statistics, *Q* can become negative. Then Eq. (36) implies that it may be possible, in principle, to make a mode arbitrarily narrow, achieving arbitrarily fine spatial resolution in a corresponding experiment, and at the same time have arbitrary small “average variance”, ${\overline{G}}_{2}-{({\overline{G}}_{1})}^{2}$.

It is instructive to look at the specific values that a two-dimensional analogue of the NRU, Eq. (36), implies for the lower limit of the product of the average degree of fourth-order coherence, ${\overline{G}}_{2}/{({\overline{G}}_{1})}^{2}$, and the width of the mode in the “classical” case (Poissonian statistics). For that, consider an experiment where measurements are performed by a two-dimensional position-sensitive detector located near the origin of coordinates in the plane $({{\bf{r}}}_{\perp },z=0)$ and a mode has Gaussian spatial distribution in the detector plane, ${f}_{k}({{\bf{r}}}_{\perp },0)={(2\pi {\sigma }^{2})}^{-1}\,\exp [\,-\,|{{\bf{r}}}_{\perp }{|}^{2}/(2{\sigma }^{2})]$. The corresponding two-dimensional version of Eq. (36) similarly follows from Eq. (9) with *d* = 2. Compared to Eq. (36), we need only replace the relative volume with the relative area, ${\Delta }_{r}^{2}[{G}_{1}(z=0)]/{L}^{2}$, and the constant ${C{\prime} }_{3}$ with ${C{\prime} }_{2}=4/(9\pi )\cong 0.14$. Now consider the two-dimensional version of Eq. (35) for Poissonian statistics, $(\langle {n}_{k}^{2}\rangle -\langle {n}_{k}\rangle )/{\langle {n}_{k}\rangle }^{2}=1$, and a Gaussian-shape mode. As the functional $F[E]$ is bi-invariant with respect to the scaling of the mode function and its argument, its value for a Gaussian mode is independent of *σ*. If *σ* $\ll$ *L*, evaluation of the relevant integrals leads to: ${\overline{G}}_{2}/{({\overline{G}}_{1})}^{2}\cong 1/(4\pi {\sigma }^{2})$, ${\Delta }_{r}^{2}[{G}_{1}(z=0)]/{L}^{2}\cong 2{\sigma }^{2}$ and hence $F[E]=1/(2\pi )\cong 0.16 > {C{\prime} }_{2}$, regardless of *σ*. For an Epanechnikov-shaped mode, ${f}_{k}({{\bf{r}}}_{\perp },0)={(1-4|{{\bf{r}}}_{\perp }{|}^{2}/{L}^{2})}_{+}$ (where the subscript “+” denotes that ${f}_{k}({{\bf{r}}}_{\perp },0)=0$ when $|{{\bf{r}}}_{\perp }| > L/2$, cf. Fig. 3(a)) and Poissonian statistics of the photon state, one obtains the minimal possible value of $F[E]={C{\prime} }_{2}$.

For a “non-classical” example with sub-Poissonian statistics, consider photon number squeezed states^27^

38 $$|\alpha ,\theta \rangle \equiv \hat{D}(\alpha )\hat{S}(\theta )|0\rangle ,$$

where $\hat{D}(\alpha )\equiv \exp (\alpha {\hat{a}}^{\dagger }-{\alpha }^{\ast }\hat{a})$ is the unitary displacement operator,

39 $$\hat{S}(\theta )\equiv \exp [(1/2)({\theta }^{\ast }{\hat{a}}^{2}-\theta {\hat{a}}^{\dagger 2})]$$

is the squeeze operator, *α*, *θ* are complex numbers, $|0\rangle$ is the Fock vacuum, $|\theta |$ is the squeeze parameter and $\langle {n}_{k}\rangle =|\alpha {|}^{2}+|\,\sinh \,|\theta {\parallel }^{2}$. It can be shown that when $|\alpha {|}^{2}\gg \exp (6|\theta |)$, the Mandel parameter can be negative:

40 $$Q=Q(\alpha ,\theta ) < [\,-\,1+\exp (\,-\,2|\theta |)] < 0.$$

In this case, the factor, $1+Q/\langle {n}_{k}\rangle$, multiplying the constant ${C{\prime} }_{3}$ on the right-hand side of Eq. (36), is less than one, meaning that the “classical” lower limit of ${C{\prime} }_{3}$ can be overcome, albeit only by a small margin which decreases with the increasing mean number of photons in the mode.

Finally, we note that, using our earlier results^9^, it can be shown that the NRU for the spatially-averaged variance of the electric energy operator, ${\rm{Var}}[{E}^{2}]\equiv \overline{\langle {E}^{4}\rangle }-{(\overline{\langle {E}^{2}\rangle })}^{2}$, can be written in a form similar to Eq. (36), but with the right-hand side always limited from below by an absolute positive constant. This difference in the form of the NRU for the photon number and the electric energy operators is caused by the presence of vacuum fluctuations which induce a shift of the absolute lower bound of the variance of electric energy operator. Interestingly, vacuum fluctuations have been also demonstrated to induce a shift in the dispersion relations between physical observables in wave models of quantum mechanics, such as prequantum classical field theory^31^.

## Discussion

A point of central importance has been only lightly touched upon in our discussions: this is the fact, reported by many workers over many decades, that the spatial resolution of an imaging system cannot be meaningfully defined in the absence of noise^11,12,14,18,32–39^. All of the various forms of noise-resolution trade-off, as given in the present paper, may be viewed as a particular re-statement of this noise-dependent notion of spatial resolution. It would be interesting to compare (i) the noise-dependent notion of resolution as quantified by the noise-resolution uncertainty principle, with (ii) other noise-dependent methods for defining resolution, such as Fourier ring correlation^40^ or approaches based on statistical parameter-estimation theory^36^.

The lower bounds, for which the two-dimensional forms of the inequalities in Eqs. (7), (9), (14), (21) become equalities, represent limits bounding the noise-resolution properties of arbitrary images. As has been previously mentioned, such limit cases correspond to the solid line bounding the shaded areas in Figs. 3(e) and 4(d). Now, in many cases, one will have a population of pixellated images, such as a database of images of handwritten characters, stars, galaxies, quasars, stellar spectra, faces, fingerprints, x-ray radiographs or x-ray diffraction patterns. Any one of these and similar image databases will define a context-specific cloud of points which lie outside the prohibited regions of two-dimensional forms of Figs. 3(e) and 4(d). For a given image database, it may be instructive to plot the corresponding cloud of points on top of the allowed regions in Figs. 3(e) and 4(d). Such plots could serve several purposes, such as (i) surveying the degree of optimality from the perspective of the noise-resolution trade-off, of a given image database; (ii) investigating strategies to improve noise or resolution in the presence of suitable constraints such as dose, acquisition time, source size and pixel size.

Another interesting avenue for future work would be to further explore the Epanechnikov states which optimise the trade-off between noise and resolution that is inherent in all of the listed forms for the noise-resolution uncertainty principle. Both motivation and context for this suggestion, is given by the huge degree of applicability of the corresponding states of minimum uncertainty product from the perspective of the Heisenberg uncertainty principle: these are the Gaussian distributions and their generalisation as given by the coherent states^27^. Indeed, it would be scarcely hyperbole to claim that the states which minimise the Heisenberg uncertainty principle’s uncertainty product, namely the coherent states of classical and quantum field theory, pervade much of the fabric of physics in general and optical physics in particular. This suggests that the Epanechnikov states may have a deeper significance that is worthy of further investigation in the context of noise-resolution uncertainty. Along similar lines, it would also be interesting to investigate states that interpolate between the Gaussian and Epanechnikov distributions, in the sense of jointly minimising some suitable combination of noise-resolution and position-momentum uncertainty.

It would also be worth exploring the implications of the present work for the formalism of partially coherent optical fields. Several concepts of direct relevance to partially coherent optical fields have appeared in the present paper, particularly in an earlier section’s mention of ergodic stochastic processes, autocorrelation lengths etc. Given that ergodic stochastic processes underpin the modern theory of partially coherent optical fields, it would be interesting to further explore any additional connections that may exist between this theory and the noise-resolution concepts in the present paper.

## Conclusions

We have demonstrated that the width of a function and the width of its histogram cannot be made arbitrarily small at the same time. This relationship can also be stated in terms of an uncertainty relationship between the spatial resolution and the signal-to-noise ratio of a distributed measurement. In the case of statistical quantities associated with stochastic or quantum observables, for example, correlation functions of bosonic fields, the NRU can in principle be made arbitrarily small for non-classical states with sub-Poissonian statistics. We have shown that photon number squeezed states in quantum optics present an example where the classical limit for the NRU can be overcome.
